# Monitoring Intestinal Organoid–Derived Monolayer Barrier Functions with Electric Cell–Substrate Impedance Sensing (ECIS)

**DOI:** 10.21769/BioProtoc.4947

**Published:** 2024-03-05

**Authors:** Sarah Ouahoud, Francesca P. Giugliano, Vanesa Muncan

**Affiliations:** Tytgat Institute for Intestinal and Liver Research, Gastroenterology Endocrinology and Metabolism, Amsterdam UMC, University of Amsterdam, Amsterdam, The Netherlands

**Keywords:** Intestinal organoids, ECIS, Impedance, TEER, Intestinal barrier, Wounding

## Abstract

The measurement of transepithelial electrical resistance across confluent cell monolayer systems is the most commonly used technique to study intestinal barrier development and integrity. Electric cell substrate impedance sensing (ECIS) is a real-time, label-free, impedance-based method used to study various cell behaviors such as cell growth, viability, migration, and barrier function in vitro. So far, the ECIS technology has exclusively been performed on cell lines. Organoids, however, are cultured from tissue-specific stem cells, which better recapitulate cell functions and the heterogeneity of the parent tissue than cell lines and are therefore more physiologically relevant for research and modeling of human diseases. In this protocol paper, we demonstrate that ECIS technology can be successfully applied on 2D monolayers generated from patient-derived intestinal organoids.

Key features

• We present a protocol that allows the assessment of various cell functions, such as proliferation and barrier formation, with ECIS on organoid-derived monolayers.

• The protocol facilitates intestinal barrier research on patient tissue-derived organoids, providing a valuable tool for disease modeling.

## Background

The intestinal epithelium is represented as a cellular monolayer that separates the luminal content from the rest of the body [1]. Apart from enabling digestion and absorption of food, it serves as a crucial barrier for warding off potentially pathogenic microbes [1]. Damage and impairment of the gut barrier are observed through the course of various intestinal diseases such as necrotizing enterocolitis and inflammatory bowel disease [2,3].

A major boost to the in vitro modeling of the intestinal epithelial barrier came with the emergence of organoid technology. Organoids are self-organizing, three-dimensional (3D) structures that are grown in vitro from stem cells [4]. They recapitulate many structural and functional aspects of their parent organ more accurately than the commonly used two-dimensional (2D) cell lines [4]. Therefore, organoids have become a frequently used model among researchers. Since it remains challenging to study epithelial barrier formation in 3D structures, several studies cultured the intestinal organoids as a monolayer in a Transwell system [1].

The Transwell system is the most commonly used in vitro model for intestinal barrier research [5,6]. It consists of a porous cell culture insert that can be placed in a traditional cell culture well plate [5]. The gut epithelial cells are grown on the permeable membrane of the insert to create a cell monolayer with a luminal and basolateral compartment. Evaluation of barrier formation is assessed by measuring the transepithelial electrical resistance (TEER) with electrodes placed on either side of the membrane [5,6]. While measuring TEER with the typical handheld chopstick electrode epithelial voltohmmeter (EVOM) device remains the gold standard for assessing the integrity of barrier models, this technique requires removal of the cultures from the incubator to test each well individually. Such approach might disrupt the cell layers and, in addition, variations in electrode positioning could hinder reproducibility of measurement because of non-uniform electric field created by the chopstick electrodes [6].

An alternative to EVOM is electrical cell–substrate impedance sensing (ECIS). ECIS uses the same principle as the EVOM, except that the electrodes are integrated into the bottom of the well on which the cells are grown [6]. The presence of the electrodes in the wells allows cells to attach and proliferate directly on top of the electrodes, resulting in more localized and sensitive impedance measurements of the cell barrier. The ECIS enables impedance measurements at a broad scale of electrical frequencies, ranging from 62.5 Hz to 64 kHz. In addition, depending on the electrode placement and size, the ECIS can be used to determine additional properties of the epithelial cell layer such as cell attachment, migration, and proliferation [6]. Studying cell attachment, proliferation, and spreading yields insights into fundamental cellular behaviors, enhancing our understanding of basic biological processes in health and disease. Apart from monitoring cell barrier formation, the cells can be subjected to (lethal) electrical currents to inflict cellular damage [7]. The ECIS is therefore a potentially interesting cell-based system to study gut barrier formation and to screen for drugs capable of resolving cell damage and achieving higher mucosal healing rates. Here, we show that the ECIS technology can be successfully applied on gut organoid 2D monolayers.

## Materials and reagents

**Biological materials**

1. Established 3D intestinal organoid lines isolated from either fetal (gestational age 18–22 weeks) or adult tissue (see general note 1)

**Reagents**

1. Advanced DMEM/F12 (Gibco, catalog number: 12634-028)
2. GlutaMAX 100× (Gibco, catalog number: 35050-038)
3. HEPES 1 M (Gibco, catalog number: 15630-056)
4. Penicillin-streptomycin (Pen/strep) 10,000 U/mL (Gibco, catalog number: 15140-122)
5. Glacial acetic acid (VWR, catalog number: 1.00056.2500)
6. L-cysteine (Sigma-Aldrich, catalog number: C7352)
7. Collagen type I, rat tail, 5 mg/mL (IBIDI, catalog number: 50201)
8. TrypLE express (1×), phenol red (Gibco, catalog number: 12605036)
9. Y27632 (ROCK-inhibitor) (Sigma-Aldrich, catalog number: Y0503)
10. Human IntestiCult organoid growth medium (includes 50 mL of IntestiCult^TM^ OGM human basal medium and 50 mL of organoid supplement) (Stemcell Technologies, catalog number: 06010)
11. Human IntestiCult organoid differentiation medium (includes 50 mL of IntestiCult^TM^ ODM human basal medium and 50 mL of organoid supplement) (Stemcell Technologies, catalog number: 100-0214)

**Solutions**

1. Ad-DF+++ (see Recipes)
2. Human IntestiCult^TM^ organoid growth medium (see Recipes)
3. Human IntestiCult^TM^ organoid differentiation medium (see Recipes)
4. 100 mM L-cysteine (see Recipes)
5. 0.1% acetic acid solution (see Recipes)
6. Collagen/0.1% acetic acid solution (see Recipes)

**Recipes**

**Media preparations**

1. **Ad-DF+++**
  Supplement 500 mL of advanced DMEM/F12 with 5 mL of Pen/strep, 5 mL of HEPES, and 5 mL of GlutaMAX.
2. **Human IntestiCult^TM^ organoid growth medium**
  Combine 50 mL of IntestiCult^TM^ OGM human basal medium with 50 mL of organoid supplement and 1 mL of Pen/strep.
3. **Human IntestiCult^TM^ organoid differentiation medium**
  Combine 50 mL of IntestiCult^TM^ ODM human basal medium with 50 mL of organoid supplement and 1 mL of Pen/strep.

**Coating solutions**

1. **100 mM L-cysteine**
  Dissolve 12.12 mg of L-cysteine in 1 mL of Milli-Q water. Pass the solution through a 0.2 µm syringe sterilization filter. The solution can be kept in the fridge for up to two weeks. Dilute the stock solution 10× in sterile Milli-Q water before the start of the experiment.
2. **0.1% acetic acid solution**
  Add 25 µL of acetic acid to 25 mL of Milli-Q water. Pass the solution through a 0.2 µm syringe sterilization filter.
3. **Collagen/0.1% acetic acid solution**
  Wells should be coated with 10 µg collagen/cm^2^. Each well has a growth area of 0.8 cm^2^. For 17 wells (16 wells + 1 extra), add 27.2 µL of collagen type I to 5,072.8 µL of 0.1% acetic acid solution.

**Laboratory supplies**

1. Applied BioPhysics ECIS 8W1E array (Ibidi, catalog number: 72001)
2. Applied BioPhysics ECIS 8W10E array (Ibidi, catalog number: 72010)
3. 15 mL tubes (Falcon, catalog number: 352096)
4. 0.2 µm syringe sterilization filters (Sarstedt, catalog number: 83.1826.001)
5. 15 mL tubes (Falcon, catalog number: 352096)
6. 200 µL pipette tips (Sapphire, catalog number: 775355)
7. 1,000 µL pipette tips (Sapphire, catalog number: 777355)

## Equipment

1. ECIS^®^ Z-Theta instrument (Applied Biophysics, catalog number: 71617)
2. 16-well array module housed in incubator including 8W test array (Applied Biophysics, catalog number: 71612)
3. Cell culture equipment (centrifuge, incubator, pipets, sterile hood, etc.)

## Software and datasets

1. ECIS Zθ software (Applied Biophysics)
2. (Optional) Excel
3. (Optional) GraphPad Prism

## Procedure

Conduct all cell and array manipulations within a sterile environment using a laminar flow cabinet.

- **Preparation of the 16-well station**
  1. Place the ECIS 16-well station in an incubator at 37 °C at least one day before the experiment to prewarm the array holder (Figure 1A ).
  2. Add water to the incubator's water reservoir to prevent the wells from drying out.
  **Figure 1.**
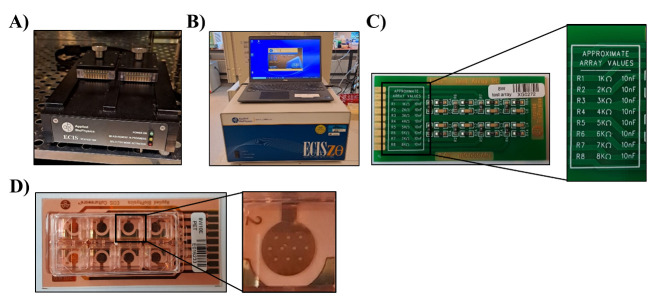
Electrical cell–substrate impedance sensing (ECIS) setup. A. The 16-well station is placed in the incubator at 37 °C. B. The ECIS^®^ Z-Theta instrument, including laptop, is placed in close proximity to the incubator holding the 16-well station. C. The 8W test array is used to calibrate the ECIS. Running the test array should provide measurements with values (close-up picture) mentioned on the array. D. The 8W ECIS arrays hold eight wells, with the 8W1E having one electrode per well and the 8W10E (close-up picture) having 10 electrodes.
- **Preparation of the arrays**
  The choice of array depends significantly on the specific scientific question and the type of cells involved. Choices for array selection include the 8W1E array for wound healing studies due to enhanced sensitivity to cell motion–induced resistance fluctuations. Multielectrode arrays, such as the 8W10E, average signals over several electrodes and are more suitable for studying cell proliferation and barrier formation by minimizing bias from uneven cell distribution.
  1. Prepare the 100 mM L-cysteine stock solution and dilute the stock 10 times in sterile Milli-Q water. For two arrays, 3,500 µL of 10 mM L-cysteine solution is needed. Therefore, add 350 µL of 100 mM L-cysteine solution to 3,150 µL of sterile Milli-Q water. See general note 2.
  2. Add 200 µL of the 10 mM L-cysteine solution to each well.
  3. Incubate the arrays for 30–45 min at room temperature (RT).
  4. Wash the wells two times with 300 µL of sterile Milli-Q water.
  5. Add 300 µL of the Collagen/0.1% acetic acid solution to each well.
  6. Incubate arrays for 60 min at RT.
  7. Wash the wells two times with 300 µL of sterile Milli-Q water.
  8. Add 400 µL of Ad-DF+++ to each well.
- **Calibration of the station and start of baseline measurements**
  1. Turn on the computer and the ECIS Zθ machine (Figure 1B). See general note 3.
  2. Open the ECIS software.
  3. Place the test array in the station, click *Setup*, and check the connection (Figure 1C and Figure 2A). If the connection is good, the wells are given a green color; the ones with a bad connection are shown in red. Reinsert the array and click *Setup* until a good connection is achieved for all wells (Figure 2B).
  4. Select the RC test array in the *array type* pop-up screen.
  5. Run the check for the test array (Figure 2C).
  6. If the RC test is OK and correct values are shown, as mentioned on the test array, you can continue using the ECIS for your experiment (Figure 1C and 2D). Contact the manufacturer if the values are incorrect.
  7. Place the array(s) with the medium in the 16-well station and perform a *check*. If electrodes have been properly stabilized with the L-cysteine, the 8W1E and the 8W10E should give values of approximately 5 nF and 50 nF, respectively. See general note 4.
  8. Select array type.
  9. Choose the *Multiple Frequency/Time (MFT)* or another mode (Figure 2C). See general note 5.
  10. Click on *Start* and enter the file name to start the run (Figure 2C ).
  11. Measure the baseline for at least 30 min. Continue with harvesting the cells during this step.
- **Preparation of cells**
  1. Remove the culture medium from the organoids and replace it with 0.5 mL of cold Ad-DF+++ for each well in a 24-well plate or 1 mL for each well in a 12-well plate.
  2. Pipette several times up and down with a p1000 pipette to disrupt the Matrigel and collect the Ad-DF+++ with the organoids in a 15 mL tube.
  3. Centrifuge at 200× *g* for 5 min at 4 °C to remove the Matrigel.
  4. Take off the supernatant and add 1–3 mL of TrypLE, depending on pellet size, to the 15 mL tube. Resuspend the cells in the TrypLE and incubate the 15 mL tube for 5–7 min in a water bath at 37 °C. See general note 6.
    **Figure 2.**
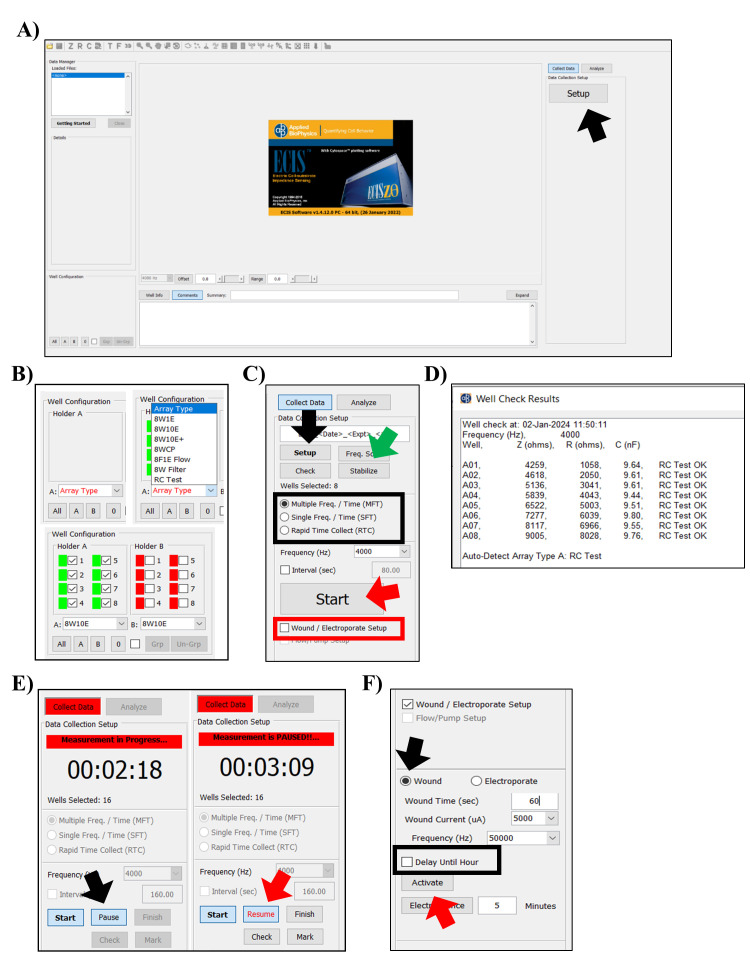
Electrical cell–substrate impedance sensing (ECIS) software. A. Upon opening the ECIS software, click *Setup* (black arrow) in the right window to measure the impedance of the device and detect an open circuit. B. Arrays/wells that have been properly connected are indicated in green in the left-bottom window of the screen, whereas arrays or wells with an open circuit are shown in red. C. Readjust the placement of the array and select *Setup* (black arrow) again to confirm correct placement of the array. By selecting *check* (button underneath *Setup*), measurements of the test array will be made using the default frequency of 4,000 Hz. If electrodes were not properly stabilized after the L-cysteine treatment, an additional stabilization step can be performed with the ECIS by selecting *stabilize* (green arrow). The same right pane holds options for the acquisition mode (outlined with a black square), the *Wound/Electroporate Setup* function (outlined with a red square), and the *Start* (red arrow) button. D. Measurements performed by selecting *Check*, e.g., results from the test array, are shown on a pop-up screen. E. Data acquisition is halted by selecting *Pause* (black arrow) and can be resumed by clicking on *Resume* (red arrow). F. After selecting *Wound/Electroporate Setup*, additional settings emerge in the bottom-right pane. Select *Wound* (black arrow), adjust the wound settings, and click *Activate* (red arrow) to perform the wounding. Wounding can be postponed to a later time point by selecting *Delay Until Hour* (outlined with a black square).
  5. Resuspend the cells vigorously for 15–20× with a p1000 pipette to generate a single-cell suspension. Adding a 200 µL tip on top of the 1,000 µL tip greatly improves the mechanical disruption of the organoids. No cell clumps or organoid structures should be visible with the naked eye. See general note 7.
  6. Inactivate the TrypLE with 10 mL of Ad-DF+++.
  7. Spin the cells at 340× *g* for 5 min at 4 °C. Remove the supernatant.
  8. Resuspend the cells in organoid growth medium (OGM) supplemented with ROCK-inhibitor (1:1,000) and perform a cell count.
  9. Prepare the single-cell suspension in the range of 150,000–300,000 cells per well. Adjust the volume with OGM supplemented with ROCK-inhibitor.
  10. Pause the baseline measurements and take the array out of the incubator (Figure 2E).
  11. Remove the media in the array.
  12. Add 400 µL of the homogenized cell suspension to each well. Do not forget to include an empty control.
  13. Put the array back in the 16-well station and resume the measurements (Figure 2E). See general note 8.
- **Experiment run and measurements**
  The frequency-selection ECIS experiments rely on the research goal and the characteristics of the cells under investigation. In resistance analysis, the 4,000 Hz frequency is used as the default setting, as this frequency is regularly used due to its relevance in capturing subtle changes associated with cell behavior and barrier function, particularly in the context of tight junction formation and integrity. However, the optimal frequency may vary, depending on the cell type and the specific experimental conditions. The frequency scan performed with the ECIS software on fetal and adult intestinal organoids showed that capacitance measurements should be analyzed at 64 kHz, whereas resistance should be analyzed at 500 Hz (Figure 3A, 3B). The capacitance and resistance data shown in this protocol paper are therefore measurements performed at a frequency of 64 kHz and 500 Hz, respectively.
  **Figure 3.**
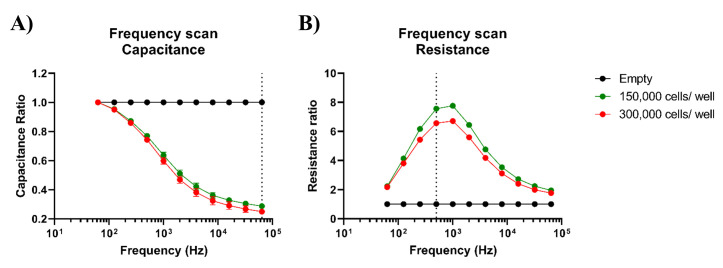
Optimal alternating current (AC) frequencies for analysis of intestinal organoid monolayers. The frequency displaying the greatest difference between cell-free (empty) and cell-covered conditions is deemed ideal for subsequent analysis. A. Capacitance data from fetal organoids grown on organoid growth medium (OGM), displayed as cell-to-cell-free ratios, exhibits a maximum response at 64 kHz (dotted line). B. However, 500 Hz (dotted line) or 1 kHz seem to be the optimal frequencies for analysis of resistance data.
  Compared to cell lines, much higher organoid cells numbers are needed to reach confluence shortly after seeding. As an example, approximately 50,000 CaCo2 cells are needed to obtain a monolayer within 24 h. ECIS capacitance measurement, which indicates electrode coverage, shows that if you seed two different organoid cell numbers per well, 150,000 or 300,000, no monolayers are formed within the first 30 h after seeding (Figure 4A–4C). After approximately 60 h, a monolayer has formed in the wells with 300,000 cells per well, and an additional 30 h is needed before a monolayer has formed in the wells with 150,000 cells per well (Figure 4D–4E). ECIS resistance measurements (500 Hz) correlate inversely with the capacitance measurements, showing that the transepithelial resistance is increasing while the monolayer is forming (Figure 4F–4J). Approximately 300,000 cells per well are needed for human intestinal organoids to reach confluence within three days, but this could differ slightly per donor depending in its growing rate. Perform a pilot experiment to determine the optimal cell number that fits your research question.
  **Figure 4.**
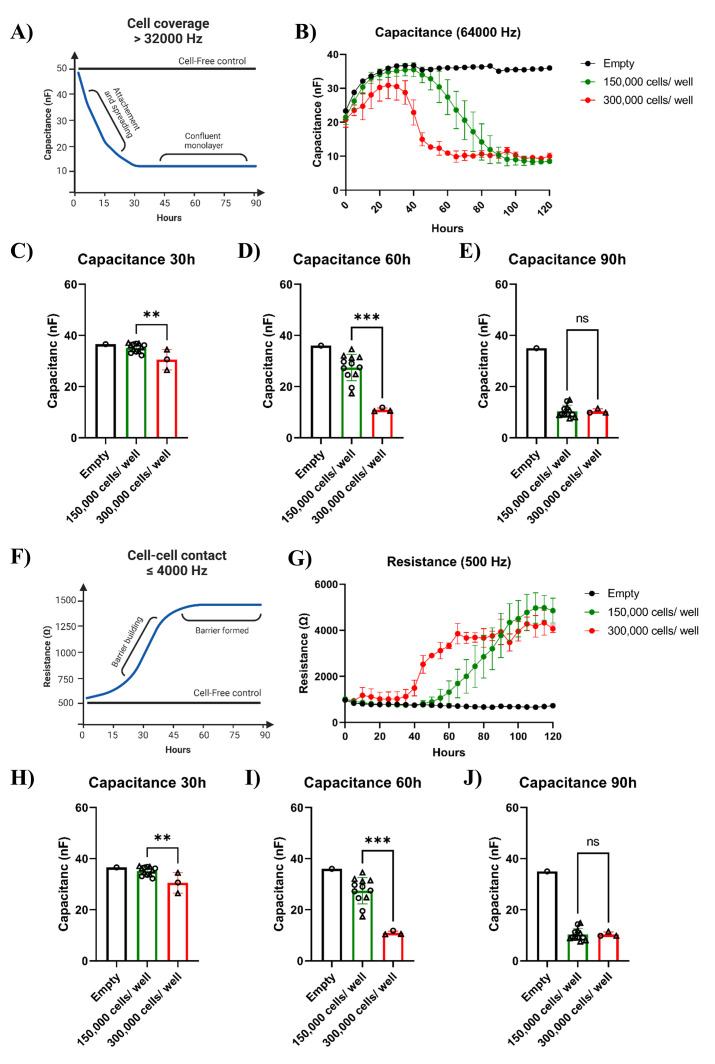
Barrier formation in 2D intestinal organoid–derived monolayers. Intestinal organoid cells from the same fetal tissue culture were seeded in two different ECIS 8W10E arrays (indicated in the graph as round or triangle shaped). A. As cells proliferate and cover the electrode surfaces, electrical current is impeded. The capacitance measured during cell proliferation is inversely correlated with surface coverage until a complete monolayer has formed. B–E. The capacitance measured at 64 kHz (indicative of electrode coverage) indicates that a full monolayer was achieved after 60 and 90 h when 150,000 (n = 12) or 300,000 (n = 3) cells were seeded, respectively. F. A significant increase in resistance is observed as the monolayers form. G–I. Resistance continues to increase and reaches a plateau at 4,000 Ω when measured at 500 Hz (indicating intact cell barrier). Bars represent mean ± SD. P < 0.05 (*), < 0.01 (**), < 0.001 (***), and < 0.0001 (****) as determined with an unpaired *t*-test.
- **Changing the medium during an experiment**
  1. Click on *Pause* to halt data acquisition. The experimental clock will continue to run.
  2. Take the array out of the holder and change the medium under a laminar flow bench. Change the medium to medium without ROCK-inhibitor 2–3 days after seeding and continue to refresh medium every 2–4 days. See general note 9.
  3. Return the array to the holder and click *Check* to check if arrays were placed back correctly.
  4. Wait for 5–10 min before resuming the measurements, as temperature changes affect the measurements.
  5. Click *Resume* to restart data acquisition. The measurement software will include a time mark in the data set. See general note 10.
- **Differentiation of cells**
  If the research question requires the cells to reflect the mature intestine and the monolayer to contain all major cell types present within tissues, an additional differentiation step might be performed [8]. By switching the culture medium from OGM to organoid differentiation medium (ODM) after the gut monolayer has been formed, an additional increase in the transepithelial resistance is observed (Figure 5). Gut cells cultured in ODM contain physiologically relevant properties of differentiated cells, such as nutrient absorption and expression of digestive enzymes and tight junction proteins.
  **Figure 5.**
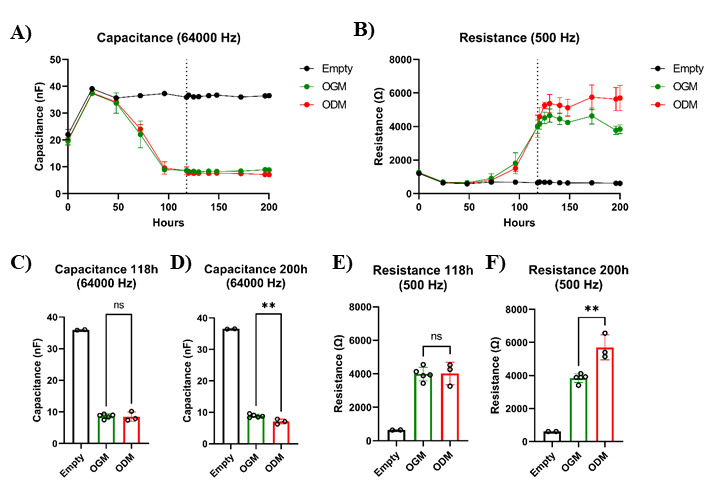
Organoid cell differentiation results in increased resistance values. Intestinal organoid cells from adult tissue were seeded into 8W10E ECIS arrays and cultured in IntestiCult organoid growth medium (OGM). After 118 h, when the capacitance plateau phase was reached but resistance values were still increasing (indicated by the dotted line), cells were further cultured in either OGM (n = 5) or organoid differentiation medium (ODM) (n = 3). A. Capacitance measurements showed significant changes between cells cultured in OGM or ODM. B) Resistance measurements, on the other hand, revealed that resistance continued to increase in cells cultured in ODM. C–F. The capacitance data of 118 h and 200 h indicate that a slight but significant change occurred in how the cells cover the electrodes, suggesting that the increase in resistance can be attributed to the formation of a tighter barrier by differentiated cells. Bars represent mean ± SD. P < 0.01 (**) as determined with an unpaired *t*-test.
- **Wound-healing assay**
  Damage to the intestinal epithelial barrier is observed in a number of gut diseases. Once a stable monolayer has formed, the ECIS can be used to inflict cellular damage by subjecting the cells to a lethal electrical current. The electrical current specifically kills the cells on top of the small gold electrodes, which subsequently die and detach from the electrode, creating a wound that is healed by neighboring cells that have not been submitted to the current [7]. This function allows for the screening of drugs that might provide significant clinical benefit by improved or faster wound healing.
  Determining the appropriate settings for the specific cell type is crucial for electrical wounding. If the wounding duration is too brief, it may lead to inadequate cell removal, while excessively long or harsh wounding can potentially damage the electrodes. For the wound-healing assays, 8W10E confluent intestinal epithelial monolayers derived from organoids were subjected to different currents. While applying a current of 4,000 μA and a frequency of 50,000 Hz for 30 s is capable of inducing enough cell damage for the cells to break their tight junction, the capacitance data shows that the current is not strong enough to kill all the cells present on the electrode. However, successful wounding on organoid-derived monolayers is achieved after applying a current of 5,000 μA and a frequency of 50,000 Hz for 60 s (Figure 6 ).
  **Figure 6.**
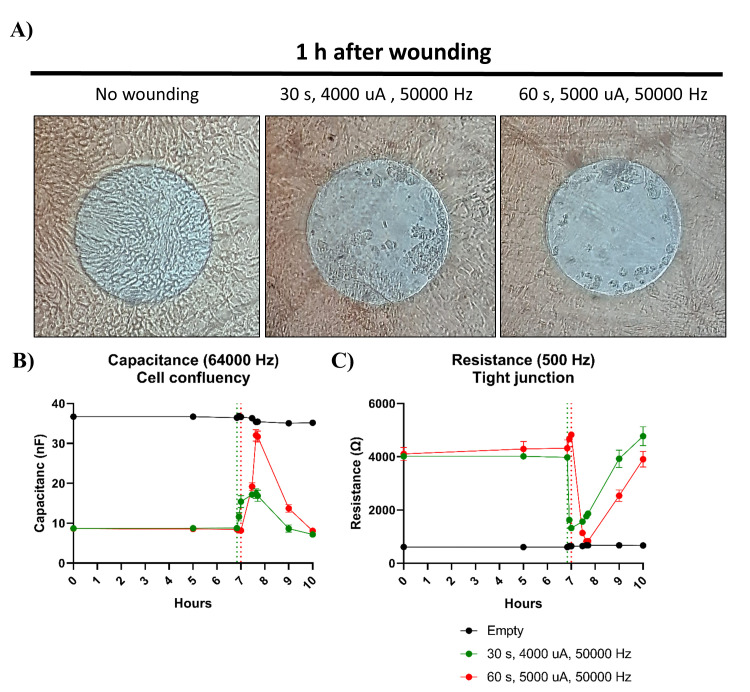
Wounding of 2D organoid monolayers. Monolayers formed by adult organoids were grown in organoid growth medium (OGM). A. Applying a current of 4,000 μA and a frequency of 50,000 Hz for 30 s was insufficient to eliminate all the cells present on the electrodes of an 8W10E array, whereas almost no cells were present when a current of 5,000 μA and a frequency of 50,000 Hz was used for 60 s. Images are representative pictures of the conditions. B–C. Capacitance and resistance measurements also demonstrated that successful wounding was only achieved when a current of 5,000 μA and 50,000 Hz was applied for 60 s, as the capacitance values did not drop to the levels observed in the empty control when a current of 4,000 μA and 50,000 Hz for 30 s was used. Dotted lines indicate time of wounding. Bars represent mean ± SD of two replicates.
  1. Select the wells you want to subject to wounding.
  2. Click the *Wound/Electroporate Setup* box (Figure 2C ).
  3. Select *Wound* (Figure 2F).
  4. Adjust the baseline settings for the wound time (s), wound current (μA), and frequency (Hz) (Figure 2F). See general note 11.
  5. You can delay the wounding to another time by selecting the *Delay until hour* (Figure 2F).
  6. Click *Activate* to perform the wounding.

## Data analysis

1. Navigate to *File*.
2. Click on *Export data*.
3. Choose *All data* or *Selected wells/time*.
4. Select either *To Excel* or *Graph data*. Use the exported data in Excel for statistical analysis and to generate graphs.

## Validation of protocol

The protocol described in this paper has undergone a meticulous optimization process through a series of experiments to ensure its effectiveness. For this protocol paper, several smaller experiments were conducted to illustrate crucial findings that guarantee a successful experiment. These smaller experiments aimed to highlight key insights and refine procedural details. Multiple replicates were carried out for each experimental condition, with careful consideration given to the distribution of replicates across two arrays. This approach not only validates the protocol's robustness but also ensures its adaptability and reliability across various conditions.

This protocol has been validated and used in the following research article: ten Hove et al. [9].

## General notes and troubleshooting

**General notes**

1. This protocol might potentially be applied, with or without minor adjustments, for human organoids derived from various tissue types.
2. Stabilizing the ECIS arrays with L-cysteine is crucial for improving electrode performance, promoting enhanced cell attachment, and sustaining a biocompatible environment. This approach guarantees that impedance measurements derived from ECIS arrays yield reliable data.
3. A detailed protocol on how to use the ECIS has been published by Szulcek et al. [10] and Anwer et al. [11].
4. If some of the wells have not reached acceptable stabilization, an alternative way to clean the electrodes can be performed using the ECIS electrical stabilization method. If the ECIS electrical stabilization method is used, you have to wait 30 min before running baseline measurements. More information about this function can be found in the online manual of the manufacturer.
5. The Multiple Frequency Time (MFT) program automatically measures selected wells across various AC frequencies and is recommended for various cell-related assays with compound effects observed over periods longer than 15 min. The Single Frequency Time (SFT) mode is suitable for rapid data acquisition at a single frequency.
6. If an incubation step of 7 min and subsequent mechanical disruption proves insufficient to obtain a single-cell suspension, the incubation step can be prolonged by an additional 1–3 min. However, do not leave the organoids for longer than a total of 10 min in TrypLE, as this affects the viability of the cells. Resuspend again after the second incubation step.
7. It is crucial to obtain a homogenous single-cell suspension, as cell clumps tend to grow as organoids after seeding. This might influence the resistance and capacitance measurements.
8. You can start the measurements directly after seeding the cells if you are interested in proliferation and barrier formation. However, if you are interested in the wounding function of the ECIS, you can put the arrays in the incubator and transfer them to the 16-well station to perform the wounding when a stable monolayer, as determined through microscopic assessment, has formed.
9. Be careful to not damage the monolayer while removing and adding the medium. Tilt the upper side of the plate slightly up and to the left (or right) so that the medium will gather into one corner of the well. Remove the medium by placing the tip in the corner where the medium has gathered. Add 400 µL of medium per well by gently pipetting the medium down the wall of the well.
10. Marks for pausing (green) and wounding (red) actions are shown as vertical dashed lines at the time points on which they were performed.
11. Wounding settings might differ between different array types. A pilot experiment to determine the optimal wounding conditions is therefore needed if another array type is used.
12. Experimental conditions might influence the susceptibility of the organoids to the wounding settings. A pilot experiment to determine the optimal wounding conditions might, therefore, be needed if cells are, for example, grown on ODM or exposed to a compound that affects cell viability.

**Troubleshooting**

Problem 1: Large variation in measurements between wells and/or abnormal high background signals during baseline measurements.

Possible cause: Electrodes have not been properly stabilized.

Solution: Always ensure that electrodes have undergone adequate stabilization before initiating baseline measurements. An extra stabilization step may be conducted using the ECIS electrical stabilization method. Alternatively, you may proceed with cell seeding, as cells also possess the capability to clean the electrodes. However, this process requires several hours, and measurements taken during this period may exhibit unusual patterns.

Problem 2: Large variation in measurements between wells.

Possible cause: Organoids are not properly converted to a single-cell suspension and grow as clumps on the electrodes.

Solution: Carefully check your single-cell suspension for the presence of organoid structures. Continue disrupting the cells if organoid structures can still be observed with the naked eye. Alternatively, include more replicates per condition to account for well variability.

Problem 3: Cells grow as cell clumps on the array.

Possible cause: Organoids are not properly converted to a single-cell suspension.

Solution: Carefully check your single-cell suspension for the presence of organoid structures. Continue disrupting the cells if organoid structures can still be observed with the naked eye.

Problem 4: Cells do not survive after seeding.

Possible cause: Cells were kept too long in TrypLE or were cultured in medium without ROCK-inhibitor.

Solution: Do not leave the cells for longer than 10 min in TrypLE and do not forget to add ROCK-inhibitor to the medium.
